# High-mobility group protein B1 derived mutant peptide mB Box-97 inhibits the formation of neutrophil extracellular traps

**DOI:** 10.3389/fimmu.2025.1565252

**Published:** 2025-04-22

**Authors:** Kunal R. More, Aishwarya Devaraj, Frank H. Robledo-Avila, Santiago Partida-Sanchez, Lauren O. Bakaletz, Steven D. Goodman

**Affiliations:** ^1^ Center for Microbe and Immunity Research, Abigail Wexner Research Institute at Nationwide Children’s Hospital, Columbus, OH, United States; ^2^ Department of Pediatrics, College of Medicine, Ohio State University, Columbus, OH, United States

**Keywords:** HMGB1, autoimmunity, inflammation, therapeutic, COVID-19, NET inhibition

## Abstract

**Introduction:**

Neutrophil Extracellular Traps (NETs) are vital for innate immunity, playing a key role in controlling pathogen and biofilm proliferation. However, excessive NETosis is implicated in autoimmunity, inflammatory and neoplastic diseases, as well as thrombosis, stroke, and post-COVID-19 complications. Managing NETosis, therefore is a significant area of ongoing research. Herein, we have identified a peptide derived from HMGB1 that we have modified via a point mutation that is referred to as mB Box-97. In our recent study in a murine lung infection model, mB Box-97 was shown to be safe and effective at disrupting biofilms without eliciting an inflammatory response typically associated with HMGB1. Here we show that the lack of an inflammatory response of mB Box-97 is in part due to the inhibition of NETosis of which we investigated the mechanism of action.

**Methods:**

mB Box-97’s anti-NETosis activity was assessed using human neutrophils with known NET inducers PMA, LPS, or Ionomycin. Additionally, mB Box-97’s binding to Protein Kinase C (PKC), in addition to downstream effects on NADPH oxidase (NOX) activation, Reactive Oxygen Species (ROS) generation and thereby NETosis were assessed.

**Results:**

mB Box-97 significantly inhibited NETosis regardless of the type of induction pathway. Mechanistically, mB Box-97 inhibits PKC activity likely through direct binding and thereby reduced downstream activities including NOX activation, ROS production and NETosis.

**Conclusions:**

mB Box-97 is a promising dual acting therapeutic candidate for managing NET-mediated pathologies and resolving biofilm infections. Our results reveal that PKC is a viable target for NETosis inhibition independent of NET inducer and worthy of further study. These findings pave the way for a novel class of therapeutics aimed at controlling excessive NETosis, potentially offering new treatments for a range of inflammatory and immune-related diseases.

## 1 Introduction

Neutrophils are a key component of the innate immune system, serving as a primary defense against pathogens including bacteria, fungi, and viruses (1, 2). Through the process known as NETosis, polymorphonuclear leukocytes (PMNs) release decondensed chromatin, decorated with antimicrobial proteins called Neutrophil Extracellular Traps (NETs) (3, 4). These released DNA tendrils can either entrap free-living pathogens or cordon off communities of microorganisms (e.g., biofilms). In the former case, the DNA tendrils act as conduits to increase the local concentration of the bound antimicrobials to enhance trapped pathogen killing, the *raison d’etre* of NETs.

Formation of NETs can be triggered by diverse stimuli, most of which are receptor mediated. Key receptors include Pattern Recognition Receptors such as Complement Receptors (CRs), Toll-Like Receptors (TLRs), Fc receptors (FcRs), chemokine receptors and other neutrophil receptors such as angiotensin-converting enzyme 2 (ACE2) (1). Activation of these receptors typically triggers the PKC-Raf/MERK/ERK pathway, which in turn activates myeloperoxidase (MPO), Neutrophil Elastase (NE), and protein-arginine deiminase type 4 (PAD4), as well as key components of nicotinamide adenine dinucleotide phosphate (NADPH) oxidase (NOX) (1, 4).

Activation of NOX is achieved through phosphorylation of its key subunits, such as p47^phox^ by PKC (5–7). Indeed, p47^phox^ deficient B lymphoblasts (8) fail to form an active NOX assembly and, as a consequence, lack a Reactive Oxygen Species (ROS) burst. This ROS burst is critical not only for bacterial killing by PMNs but also for initiating NETosis (6). ROS facilitates the permeabilization of various cellular membranes, including those of azurophil granules that hold MPO and NE amongst other serine proteases. These proteases degrade cytoskeletal elements which contributes to NETosis (3). Additionally, they migrate to the nucleus, where they degrade lamin and histones, which leads to chromatin decondensation and breakdown of the nuclear envelope. Concurrently, PAD4, which can also be activated by a rise in Ca^2+^, gets transferred from the cytoplasm into the nucleus where it catalyzes citrullination of histones leading to chromatin decondensation to facilitate one dimensional movement of bound antimicrobials on the DNA strands/tendrils (3, 9, 10).

NETosis inducers are widespread. One of the most extensively studied inducers is phorbol 12-myristate 13-acetate (PMA), which induces NET formation via activation of PKC (11). Microbial infections can also provoke NETosis through the activation of PKC (1, 12, 13). Engagement of TLRs by various microorganisms initiates NOX-dependent NETosis, for example, recognition of *Streptococcus suis* serotype 2 (SS2) by TLR2 and/or TLR4 (1). CD11b/CD18, CD14, dectin-1, and TLRs are key in recognizing fungal mannans which leads to the activation of PKC, and ultimately NOX-dependent NETosis (1, 14). In contrast, the Ca^2+^ ionophore A23187 triggers NETosis via an alternative pathway independent of ROS generation (1). NETs produced by each of these stimuli are proteolytically active, kill bacteria, and are comprised primarily of chromosomal DNA (1, 4, 13).

Despite their crucial defensive roles, excessive NETosis and NETs can contribute to various pathologies. NET-associated molecules can become autoantigens, triggering autoimmune responses. For example, citrullinated proteins such as histones and vimentin serve as neoepitopes for anti-citrullinated protein antibodies (ACPA) in rheumatoid arthritis (RA). NET-derived extracellular nucleic acids and dsDNA are the targets of systemic lupus erythematosus (SLE). NET-associated MPO and proteinase 3 (PR3) enzymes are major autoantigenic targets of anti-neutrophil cytoplasmic antibody (ANCA)-associated vasculitis (AAV) (1, 15). When these molecules escape from NETs, they can induce uncontrolled inflammation and tissue damage. Thus, excess NETs may cause uncontrolled inflammatory responses which leads to tissue pathology (1, 4, 16). In some SARS-CoV-2 infections, excessive NETosis is linked with the development of acute lung injury (ALI) and acute respiratory distress syndrome (ARDS) due to the creation of the NETs-IL-1β loop. In some patients with severe COVID-19, aberrant NET formation may augment a SARS-CoV-2-induced cytokine storm (CS) and macrophage activation syndrome (MAS) (17). Angiotensin-converting Enzyme 2 (ACE2) down-regulation by SARS-CoV-2 elicits activation of the HMGB1 pathway which leads to the activation of cytokine storm-induced-ALI/ARDS (17). Platelet-derived HMGB1 released by neutrophil interaction with platelets can induce inflammation and over-stimulate NETs formation. In addition, the NETs themselves can serve as physical barriers that result in thrombosis (4, 17, 18) and obstruction to cause organ damage e.g. ischemic stroke brain injury (19) particularly when NETs are produced in excess. Consequently, developing therapeutics that regulate or reduce excessive NETosis is critical for mitigating these complications.

HMGB1 serves diverse biological functions beyond NETosis induction. HMGB1 gets internalized in endothelial cells via dynamin and RAGE (receptor for advanced glycation end products)-dependent signaling (20). Intracellularly, HMGB1 can travel to the nucleus serving as a DNA architectural protein critical for various DNA transactions. Extracellularly, HMGB1 is a proinflammatory innate immune effector that elicits PMN migration, induces NETosis, and exhibits anti-biofilm activity (21–23). Previously we showed that recombinant HMGB1 (rHMBG1) exhibits anti-biofilm activity by virtue of its ability to bind DNA that disrupts the extracellular DNA-dependent protective matrix of biofilms (23). This dual role suggests that the HMGB1’s DNA-binding functions and ability to induce NETs are interconnected: HMGB1 not only drives PMN migration and NETosis, but also inhibits biofilm proliferation, thereby linking its immune response and biofilm control activities. More recently, we have shown that this anti-biofilm activity is localized to the B Box domain of HMGB1 which coincides with the proinflammatory activity of this innate immune effector protein (24). Notably, a synthetic variant of this domain, mB Box-97_syn_, engineered with a point mutation (C106S) to diminish its pro-inflammatory properties, retains its DNA-binding and anti-biofilm activities (24).

Here we show that mB Box-97_syn_, unlike its wild-type counterpart (B Box-97), strongly inhibits NETosis across different induction pathways. Specifically, mB Box-97_syn_ effectively blocks both PMA and pathogen-induced NETosis, while also partially inhibiting Ca^2+^ ionophore-induced NETosis. Mechanistically, this inhibition is linked to the suppression of PKC activity, thus reducing the activation of the NADPH oxidase complex, the Nox-2-dependent pathway of NETosis. Finally, we discuss how our findings explain the native mechanism of HMGB1 NET induction as well as identify a NETosis Achilles heel which could lead to a new class of therapeutics that suppress excessive NETosis.

## 2 Methodology

### 2.1 Isolation of neutrophils from normal human blood

Blood was collected (20 mL per subject) in heparinized tubes (BD, Franklin Lakes NJ). Human neutrophils were isolated from blood using the EasySepTM Human neutrophil isolation kit (StemCell Technologies, Inc., 17957).

### 2.2 Recombinant and synthetic proteins (human rHMGB1, A Box, B Box-87, B Box-97, mB Box-97rec, AB Box, mB Box-97-His, mB Box-97_syn_)

N-terminal 6-His-mB Box was cloned into pET-15b. Recombinant proteins without tags were generated using the IMPACT kit (New England Biolabs). All constructs were confirmed by DNA sequencing. Proteins were purified to >95% purity using DEAE HiTrap and Heparin Sepharose columns and quantified using the Pierce BCA Protein Assay kit and SDS-PAGE with silver stain (ThermoFisher, 24612) with known protein standards. All the recombinant proteins were tested for the presence of endotoxin using Pierce™ Chromogenic Endotoxin Quant Kit (Thermo Scientific™, A39552S) and endotoxin free preparations were used for the further studies. Proteins are also confirmed by Western blot analysis. Recombinant HMGB1 (rHMGB1) was also confirmed by LC-MS/MS. 97 amino acids peptide mB Box-97_syn_ was synthesized by 179 LifeTein, LLC, 601 US Rt.206, Suite 26-463 Hillsborough, NJ 0884 (mB Box-97_syn_; LifeTein^®^, LLC; for ease of large-scale production and > to 95% homogeneity).

### 2.3 Quantitation of NETs

10^5^ PMNs per well in Hanks’ Balanced Salt Solution (HBSS) supplemented with calcium and magnesium (Fisher, 14025092) in an 8-well glass bottom chambered slide were allowed to attach for 30 minutes at 37°C. Cells were then stimulated with 100 nM PMA with or without peptides A Box/B box/AB Box/B Box-87/B Box-97/synthetic mB Box-97_syn_/mB Box-97-His (200 nM) or the peptides alone and incubated for 3-4 hours at 37°C. DNA was stained with 5×10^−6^ M SYTOX™ Green (Invitrogen, S7020) for 15 min. NETs were imaged with a Zeiss LSM 800 confocal microscope (Carl Zeiss Inc.). Cells were counted manually and NET forming cells % of the total cells present per well were plotted using GraphPad Prism.

10^5^ PMNs were treated with media alone or 100 nM PMA in the presence or absence of the above peptides or peptides alone for 3 hours. Extracellular DNA was stained with 5×10^−6^ M of SYTOX™ green (Invitrogen, S7020) for 15 minutes, and the sample fluorescence was measured at 504/523 nm with a Synergy H1 multi-mode plate reader.

### 2.4 Visualization of NET structures

1 x 10^5^ PMNs in HBSS (Fisher, 14025092) per well in 8-well glass bottom chambered slide were allowed to attach for 30 minutes at 37°C. Cells were then stimulated with 100 nM PMA, heat-inactivated NTHI (MOI=10), or 1 µM Ca2+ ionophore A23187 (Millipore Sigma, C7522) with or without mB Box-97_syn_ or B Box-97 (200 nM) and incubated for 3-4 hours at 37°C. To visualize NETs, we used a method previously described (23). Briefly, NETs were fixed in formalin, blocked with 10% normal goat serum (Fisher, 50197Z), and labeled with α-dsDNA mouse monoclonal antibody (1 μg; Abcam, ab27156), and α-neutrophil elastase rabbit monoclonal antibody (1 μg; Abcam ab131260) or anti-Histone H3 rabbit polyclonal antibody (citrulline R2 + R8 + R17) antibody (1 μg; Abcam, ab5103) or anti-Myeloperoxidase recombinant rabbit monoclonal antibody (1 μg; Abcam, ab5103) or naive rabbit and mouse isotype controls (1 μg; Abcam, ab37415 and ab18415) in 200 μl PBS for 16 hours at 4°C. NETs were washed with PBS and incubated for 1 hour with 1:200 dilution goat α-rabbit IgG conjugated to AlexaFluor^®^ 405 (Invitrogen, A11032), goat α-mouse IgG conjugated to AlexaFluor^®^ 594 (Invitrogen, A11001), and 1:500 dilution wheat germ agglutinin conjugated to AlexaFluor^®^ 488 (PMN membrane stain, Fisher scientific, W6748). NETs were imaged with a Zeiss LSM 800 confocal microscope (Carl Zeiss Inc.) and rendered with Zeiss Zen software. Using ImageJ, Z-stack projection of the images was generated by summing all the stacks, followed by brightness and contrast adjustments using the auto setting for all fluorophores to improve the image quality of the representative images presented in the manuscript. MFI values for NE/MPO/H3Cit, dsDNA, and Plasma Membrane (PM) using ImageJ.

### 2.5 Quantification of released and DNA-bound NE

A total of 5 x 10^6^ neutrophils in HBSS (Fisher, 14025092) per well were seeded into 6-well plates. Cells were treated with media alone or 200 nM PMA with or without B Box-97/mB Box-97_syn_ (1 μM) for 3-4 hours. Media from each well were collected and centrifuged at 10,000 RPM for 10 min to remove the cells or cell debris. This fraction was labeled as secreted NE. The NETs at the bottom of each well were incubated with DNAs I for 30 min, cells, and cell debris were removed by centrifugation as mentioned above and the supernatant collected was labeled as DNA-bound. Both fractions were subjected to the quantification of NE using a Human Neutrophil Elastase/ELA2 DuoSet ELISA kit (R&D systems, DY9167-05) as per the manufacturer’s instructions. Briefly, the Capture antibody was coated to the 96 well plates overnight. Samples and standards were incubated for 2 hours at RT and washed thrice with wash buffer post incubation. Each well was incubated with detection antibody for 2 hours at RT and washed trice post incubation with wash buffer. The detection antibody was probed with streptavidin-HRP by incubating at RT for 20 min. Post incubation each well was washed trice with wash buffer and incubated for 20 min (or till the color develops) at RT, the reaction was stopped with 2 N H_2_SO_4_ (stop solution) and plates were read using a plate reader.

### 2.6 Co-localization of mB Box-97

1 x 10^5^ PMNs per well HBSS (Fisher, 14025092) were stimulated with 100 nM PMA with or without recombinant mB Box-97-His with N-terminal His tag (200nM) and incubated for 3-4 hours at 37°C. NETs were probed for the peptide using 1:200 diluted 6x-His Tag Recombinant Rabbit Monoclonal Antibody (Fisher Scientific, MA5-33032) and for DNA with α-dsDNA mouse monoclonal antibody (1 μg; Abcam, ab27156). Post washing, these antibodies were detected as mentioned above. NETs were imaged with a Zeiss LSM 800 confocal microscope (Carl Zeiss Inc.) and rendered with Zeiss Zen software. Co-localization analysis was carried out using NIS-Elements AR (Nikon) with the workflow created to calculate Pearson co-localization coefficient (PCC). The PCC values were plotted using GraphPad Prism. PCC greater than 5 shows strong, between 0.3 and 0.5 shows moderate and 0 to 0.3 shows weak co-localization. 0 or below shows a negative colocalization.

### 2.7 Measurement of reactive oxygen species

PMNs were stained with highly reactive oxygen species (hROS) detection reagent Luminol as described (25). Briefly, 10^5^ PMNs were seeded into the wells of 96 well black plates and incubated for 10 min at 37°C. After the addition of 100 µM luminol (Sigma Aldrich) cells were incubated for 5 min at RT, and activated with 100 nM of PMA alone, PMA with 200 nM B Box-97/mB Box-97_syn_/mB Box-97-His or B Box-97/mB Box-97_syn_ alone, or media alone. Luminescence as a measure of generation of ROS was measured for 120 min at every 5 min interval using Synergy H1 multi-mode plate reader (Biotek, Winooski VT).

### 2.8 Western blot analysis

5 x 10^6^ isolated human neutrophils were treated with media alone (HBSS, Thermo Scientific, 14025092) or PMA (200 nM) with or without mB Box-97_syn_ or B Box-97 (1 µM) for 30min. Cells were homogenized in RIPA Lysis and Extraction Buffer (Thermo Scientific, 89901) supplemented with Halt™ Protease and Phosphatase Inhibitor Cocktail (Thermo Scientific, 78442). The concentration of total proteins was quantified using the BCA (Thermo Scientific, 23227) and, 20 µg total proteins were separated on 12% SDS-PAGE gels and electro-transferred to nitrocellulose membranes, blocked for 30 min with 5% fat free dry milk in PBST (PBS with 0.1% Tween 20) before the primary antibodies were applied. Antibodies used in Western blot were anti phospho-p47phox (Ser370) rabbit polyclonal antibody (1:500, Invitrogen, PA5-36863), rabbit monoclonal anti p47-phox (Abcam, EPR27205-231) Mouse anti-GAPDH (1:6000, Proteintech, 60004-1-Ig), HRP-conjugated anti-rabbit (1:10,000, Jackson, 111-035-003), HRP-conjugated anti-Mouse (1:10,000, Jackson, 115-035-003). After overnight incubation at 4°C with primary antibodies and washing, corresponding HRP-conjugated secondary antibodies were incubated for 2 h at RT, followed by washing. The HRP signals were detected by an enhanced chemiluminescence reagent (Thermo Scientific, PI80196) and imaged by a super-sensitive multifunctional imaging instrument (Amersham Imager 680UV, GE). The density of each protein band was analyzed using ImageJ and the density of phospho p47^phox^ was plotted post normalization with total p47^phox^ and GAPDH as Arbitrary Units (AU) using GraphPad Prism.

### 2.9 NET inactivation of bacterial killing

Inactivation of bacterial killing by NETs is measured as described previously (26). Briefly, NTHI strain 86-028NP, isolated from the nasopharynx of a child with chronic otitis media at Nationwide Children’s Hospital (27) was cultured on chocolate agar for 18-24h at 37°C in a humidified atmosphere that contained 5% CO2. NTHI was then resuspended in brain heart infusion broth supplemented with heme (2 μg/mL) and β-NAD (2 μg/mL) (sBHI) broth to an OD_490_ of 0·1. The culture was then diluted to approximately 2 × 10^5^ CFU/mL, and 200 μL of this suspension was inoculated into each well of an 8-well chambered cover-glass slide (Thermo Fisher Scientific, Waltham, MA) for 16 hours. Biofilms were washed carefully with PBS to remove non-adherent bacteria, followed by the addition of 300 ml of RPMI supplemented with 10% FBS. 1x10^6^ neutrophils were then added to the wells and treated with either: 1 µM HU_NTHI_ (positive control), 1 µM mB Box-97_syn_, or B Box-97 for 4 hours. After incubation, during which time biofilm remodeling occurs, 0.1% Triton X-100 was added to the cultures to release any intracellular bacteria from the PMNs after which all bacteria were recovered by homogenization via pipetting, followed by serial dilutions and plating (25). The percentage of reduction of the number of NTHI (total CFU) and % relative killing was calculated as indicated in the following formulas: [(CFU of each sample) ÷ (average CFU of NTHI only controls) 100 = % viable]: [% Killing = 100% - (% viable)]. The relative % killing was normalized so that the NTHI only control represented 0% killing as follows: [100% (e.g., an average of NTHI control) – (% killing of each sample) = relative % killing]. The graph plots the data of the PMNs derived from 6 healthy donors ± SEM. The statistical analysis was performed with One-way ANOVA and Dunnett multiple comparison test. (** = p < 0.01).

### 2.10 Inhibition of PKC activity

The effect of mB Box-97_syn_/B Box-97 on the activity of PKC was assessed using a PKC kinase activity kit (Enzo Life Sciences, ADI-EKS-420A) as per manufacturer instructions. Briefly, 30 µL of 2 ng PKC with or without proteins (histone H1, mB Box-97_syn_ and B Box-97) at 1uM final concentration in the activity assay buffer were added to the precoated wells with PKC substrate. The reaction was initiated using 10 µL ATP and incubated at 30°C for 90 min. After the removal of reaction reagents, each well was incubated with 40 µL of the phosphospecific substrate antibody for 60 min at RT. Each well was washed 4 times with wash buffer and 40 µL of the Anti-Rabbit IgG: HRP conjugate was added to each well, except the blank. After incubation for 30 min at RT, each wells were washed with wash buffer 4 times. 60 µL TMB substrate was added to each well and incubated at RT till the development of the color (30-60 min). The reaction was stopped by adding 20 µL of the stop solution and read at 450 nM using a plate reader. Data were normalized and plotted using GraphPad Prism.

### 2.11 Glutaraldehyde crosslinking assay

Crosslinking was performed as previously described with slight modifications (28). Briefly, PKC alone or PKC and mB Box-97_syn_ or B Box-97 at 1:2 molar ratio was incubated with 0.01% glutaraldehyde in HBSS (Thermo Scientific, 14025092) and 5ul 1 mg/mL ATP for 30 minutes at room temperature in a total reaction volume of 15 µL. Equal volumes of 2× Laemmli sample buffer were added, and the samples were separated using SDS-PAGE 20-40% gradient gel. Gels were either stained with silver stain (ThermoFisher, 24612) or transferred on the nitrocellulose paper to investigate the presence of peptide using anti HMGB1 rabbit antibody (Abcam, ab79823) which was then detected using anti HRP-conjugated anti-rabbit (1:10,000, Jackson, 111-035-003). The HRP signals were detected by an enhanced chemiluminescence reagent (Thermo Scientific, PI80196) and imaged by a super-sensitive multifunctional imaging instrument (Amersham Imager 680UV, GE).

### 2.12 Stability assessment of mB Box-97_syn_

1µg mB Box-97_syn_ was incubated with human serum or PBS for 0 hr and 24 hours. To test the stability of the peptide in neutrophil conditioned media, a total of 5 x 10^6^ neutrophils in HBSS (Fisher, 14025092) per well were seeded into 6-well plates. Cells were treated with 2 x 10^-7^ nM PMA for 4 or 16 hours. Media was collected, and centrifuged at 10,000 RPM for 10 min to remove the cells or cell debris. 1µg mB Box-97_syn_ was incubated with the conditioned media from above preparation or HBSS alone for 4, and 24 hours. Post incubations the peptide was run on a gel, transferred on the nitrocellulose paper to investigate the presence of peptide using anti HMGB1 rabbit antibody (Abcam, ab79823) as mentioned above.

### 2.13 Annexin V and propidium iodide staining

5 x 10^5^ PMNs were treated either alone with mB Box-97_syn_ or B Box-97 or stimulated with 5 x 10^-8^ M of PMA in presence or absence of mB Box-97_syn_ or B Box-97 for 1 and 3 hours, then cells were stained with anti-Annexin V-FITC (Biolegend, 640906) and propidium iodide (Biolegend, 421301), the cells were washed with HBSS and detected by flow cytometry (BD LSRFortessa). The samples were analyzed by FlowJo software v 10.10 (BD) and graphs were plotted using GraphPad Prism v 10.1.4.

### 2.14 Statistics

Graphical results were analyzed, and statistical tests were performed with GraphPad Prism 10 for all *in vitro* assays. Data from two groups were analyzed by unpaired t-tests, whereas data from multiple groups were analyzed by one-way/two-way analysis of variance (ANOVA) with Dunnett’s multiple comparisons tests. Data are presented as mean ± SEM and significance is shown as *P< 0·05, **P < 0·01, ***P < 0·001, and ****P <0·0001. All the statistical details are provided in the respective Figure legends of each Figure and includes the statistical tests used, the exact value of n, and what n represents. Each biological replicate had 3 technical replicates.

## 3 Results

### 3.1 mB Box-97_syn_inhibits NETosis by human neutrophils

**Figure 1:**
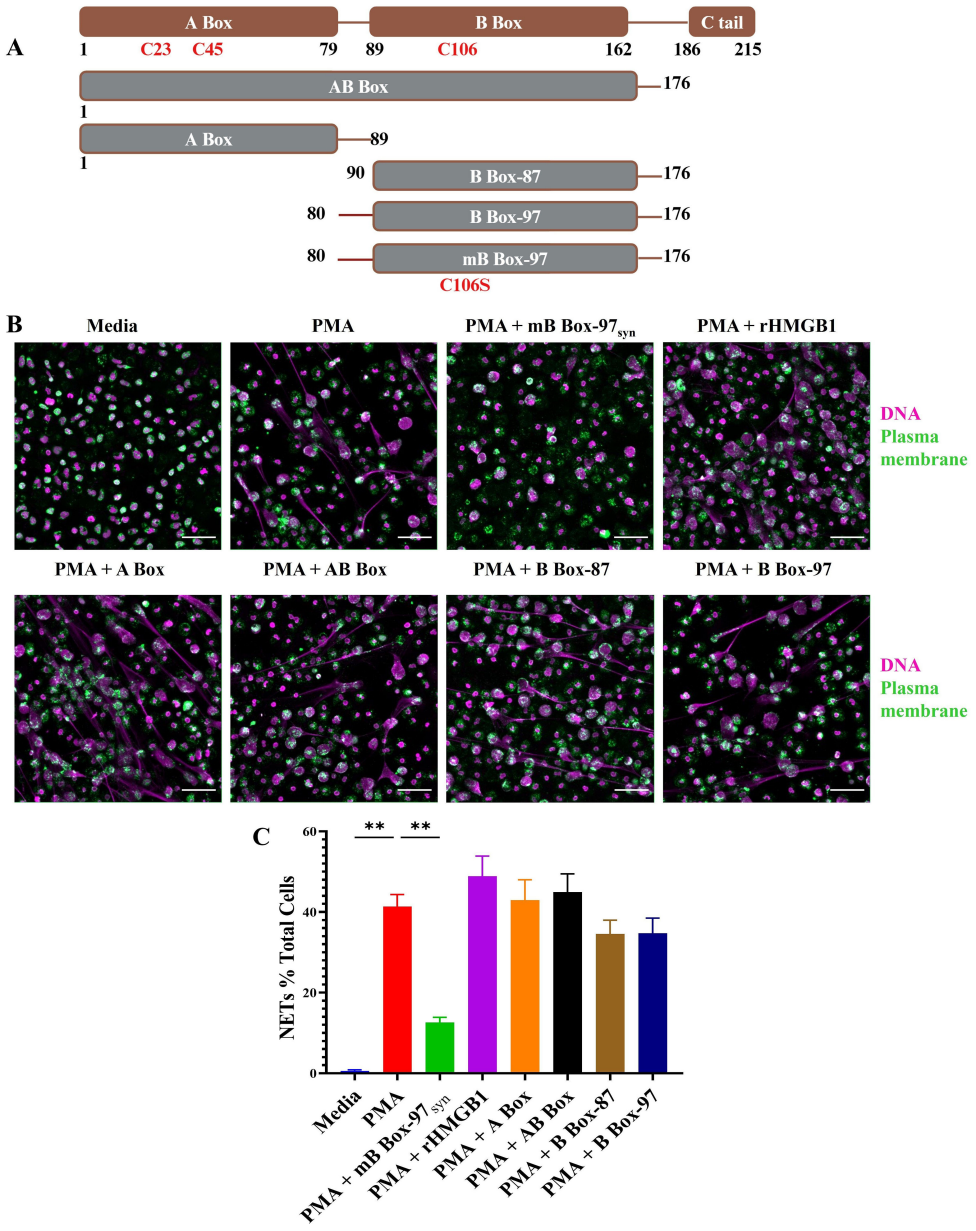
Only mB Box-97_syn_ inhibited NETosis induced by PMA. **(A)** Schematic depiction of the peptide constructs utilized in this study. Full-length HMGB1 is represented in brown, showcasing DNA binding domains A Box, B Box, and a C-terminal acidic tail. Various peptide constructs, indicated in gray with brown borders, illustrate amino acid positions from N-terminal to C-terminal. Construct names are denoted in white. Specific Cysteines/C and their corresponding amino acid numbers are denoted; in mB Box-97_syn_, C is mutated to Serine/S at position 106. **(B)** NETs were induced using PMA in the presence or absence of respective peptides or recombinant HMGB1 (rHMGB1) and stained to detect DNA (magenta) and Plasma Membrane (PM) (green). NETs were visualized using confocal laser scanning microscopy (CLSM) at 20x magnification, with representative images provided for each treatment condition out of 3 independent biological replicates (n), scale bar 50 µm. **(C)** Formed NETs were manually quantified using ImageJ, and the percentage of NET formation was calculated relative to total PMNs in each image. Mean values of n = 5 biological replicates ± Standard error of the mean (SEM) is shown. p values (ns = p > 0.05, **p < 0.01) are from a Welch and Brown-Forsythe ANOVA followed by Dunnett’s T-test.

In a previous study (24), we aimed to pinpoint the antibiofilm activity of HMGB1 by making a series of peptide constructs based of HMGB1 domains (**Figure 1A**). Here, we investigated those peptides to determine if any specifically affected known HMGB1 mediated neutrophil functions. Recombinant full-length HMGB1 (23), AB Box (amino acids 1 to 176), A Box (amino acids 1 to 89), B Box-87 (amino acids 90 to176), B Box-97 (amino acids 80 to 176 where amino acids 80 to 90 contain the positively charged linker region between A Box and B Box), and recombinant mB Box-97 and synthetic mB Box-97_syn_ (based on B Box-97 of HMGB1, NCBI-BAA09924.1 except for a single amino acid change C106S that reduces the proinflammatory effect of the B Box-97) (24) (**Figure 1A**) were each tested for their ability to induce NETosis by human PMNs (**Supplementary Figure S1**). As there were no differences in the activity of recombinant versus synthetic mB Box-97_syn_ with regards to antibiofilm activity (data not shown), to reduce any variation due to post translational modifications we used synthetic mB Box-97, mB Box-97_syn_ for all subsequent experiments unless specified. PMNs were incubated with the peptide constructs, fixed, permeabilized, and stained for DNA using SYTOX™ green. Cells were then visualized via confocal scanning laser microscopy (CSLM). mB Box-97_syn_, B Box-87, AB Box and A Box were unable to induce NETosis on their own (**Supplementary Figure S1A**). This finding was confirmed by a plate-based assay where we stained extracellular DNA with SYTOX™ green and analyzed for relative fluorescence relative to media alone (**Supplementary Figure S1B**).

We then determined whether any of these peptides affected PMA-induced NETosis. Here, PMNs were treated with PMA alone or with PMA plus each of the HMGB1-derived peptides, fixed and permeabilized. PMNs were then stained for plasma membrane and double stranded DNA and visualized via CSLM. Only mB Box-97_syn_ significantly inhibited PMA induced NETosis among all the tested constructs (**Figure 1B**). Indeed, upon quantification of PMNs that formed NETs, mB Box-97_syn_ reduced PMA-induced NETosis 3-fold compared to PMA + B Box-97 (**Figure 1C**). To ensure the lack of turnover of the peptide throughout the course of this experiment, we also evaluated the stability of mB Box-97_syn_ in conditioned media collected from the neutrophils stimulated with PMA to induce NET formation for 4 and 16 hrs. Peptides were incubated for 4 and 24 hours in conditioned media and were run on SDS-PAGE and stained to identify gross peptide degradation. As shown in **Supplementary Figure S2A** the peptides were stable after 4 hours of incubation with neutrophil conditioned media (< 5% degradation) while there was <20% degradation upon incubation for 24 hours. This suggests that mB Box-97_syn_ peptide is stable for more than 4 hours when incubated with the neutrophils forming NETs. Finally, we also tested the impact of B Box-97 and mB Box-97_syn_ on the neutrophils; there were no changes in the viability between untreated neutrophils and B Box-97 or mB Box-97_syn_ peptide treated neutrophils (**Supplementary Figure S2B**).

### 3.2 mB Box-97_syn_ inhibited PMA- and LPS-induced (NOX-dependent) NET formation and the release of NET-associated proteins

**Figure 2:**
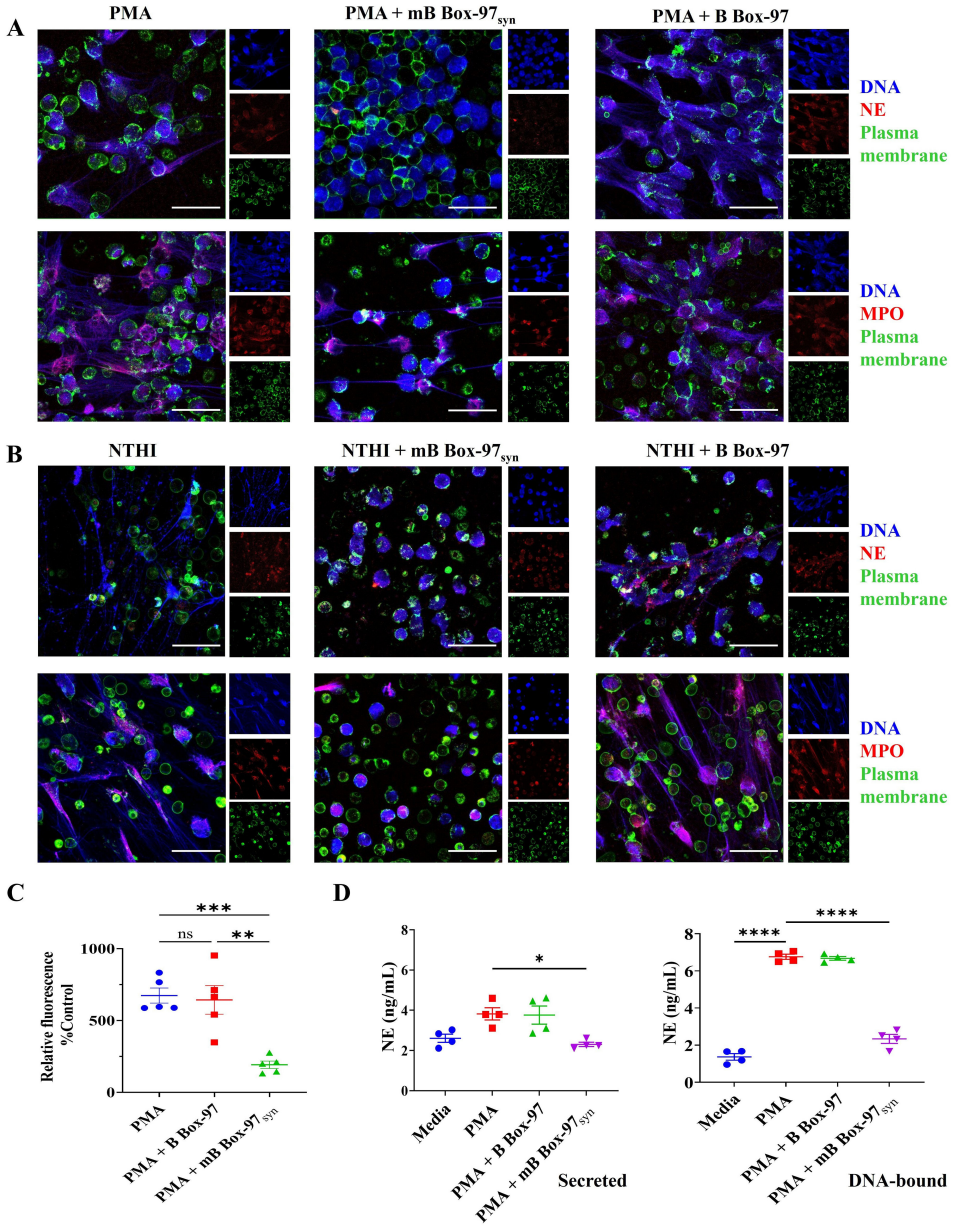
mBBox-97 inhibited both PMA- and NTHI-induced NETosis as well as the secretion of the NET-associated protein Neutrophil Elastase (NE). **(A)** The induction of NETs by PMA or **(B)** by heat-inactivated NTHI was assessed in isolated human PMNs in the presence or absence of mB Box-97_syn_ or B Box-97. NETs were stained for NET-associated proteins, namely Neutrophil Elastase (NE, upper panel) and Myeloperoxidase (MPO, lower panel) shown in red; DNA shown in blue and plasma membrane shown in green. Representative 63x magnification CLSM images for each treatment condition are shown out of 3 independent experiments (n), scale bar 50 µm. **(C)** Fluorescence of DNA released from the PMNs induced with PMA with or without the addition of mB Box-97_syn_ or B Box-97 was plotted as a percentage relative fluorescence compared to the untreated control group. Mean values of n = 5 biological replicates ± SEM are shown. **(D)** PMNs were induced to form NETs with PMA alone or PMA +/- B Box-97/mB Box-97_syn_ and NE released (secreted) into the media was collected. The NETs settled at the bottom of each well were treated with DNase I to liberate DNA-bound NE (DNA-bound). The concentration of NE was quantified using ELISA and presented as mean ± SEM, Mean values of n = 4 biological replicates ± SEM are shown. P values (ns = p > 0.05, *p < 0.05, **p < 0.005, ***p < 0.0005, ****p < 0.0001) using from a Welch and Brown-Forsythe ANOVA followed by Dunnett’s T test.

Next, we investigated the impact on the release of NET associated proteins such as NE, MPO, or citrullinated histone (H3Cit) upon inhibition of NETs using mB Box-97_syn_. Isolated human PMNs were treated with PMA or heat-inactivated nontypeable *Haemophilus influenzae* (NTHI; as a source of LPS) with or without either B Box-97 or mB Box-97_syn_ and stained for the identification of DNA, PM and NETs associated proteins using specific antibodies. NET formation was observed by comparing z-stack images captured using CSLM. mB Box-97_syn_ inhibited PMA-induced (**Figure 2A**) as well as NTHI LPS-induced NETosis (**Figure 2B**), whereas B Box-97 did not affect either (**Figure 2**). mB Box-97_syn_ showed inhibition in the release of NETs associated proteins NE and MPO (**Figure 2**) while both mB Box-97_syn_ and B Box-97 had no measurable effect on H3Cit release (**Supplementary Figure S3**). We calculated the mean fluorescence intensity (MFI) for NE/MPO/H3Cit; DNA and PM. MFI for NE/MPO/H3Cit were normalized to the MFI for DNA and PM. Upon addition of mB Box-97_syn_, MFI values for NE and MPO were significantly reduced compared to those of PMNs treated with either PMA/NTHI alone or PMA/NTHI plus B Box-97. There was no difference in the MFI for citrullinated histone in either treatment condition (**Supplementary Figure S4A, B**).

To quantify relative NETosis, we used cell impermeable DNA stain SYTOX™ Green to stain released DNA from PMNs. mB Box-97_syn_ showed significant inhibition of PMA-induced NET formation whereas B Box-97 had no significant effect (**Figure 2C**). We also investigated the release of NE into the surrounding media as well as that bound to DNA post-PMA-induced NETosis. Neutrophils were induced to form NETs and NE bound to DNA or secreted into the surrounding medium was quantified using NE-specific ELISA. NE, when present in a free circulating state, contributes to chronic inflammation while the DNA-bound NE in NETs degrades plasminogen, reduces plasmin formation, and decreases fibrinolysis (29, 30). mB Box-97_syn_ treatment significantly reduced the free NE (secreted), as well as that bound to DNA (DNA-bound) (**Figure 2D**).

### 3.3 mB Box-97_syn_ partially inhibited Ca^2+^-mediated NETosis

**Figure 3:**
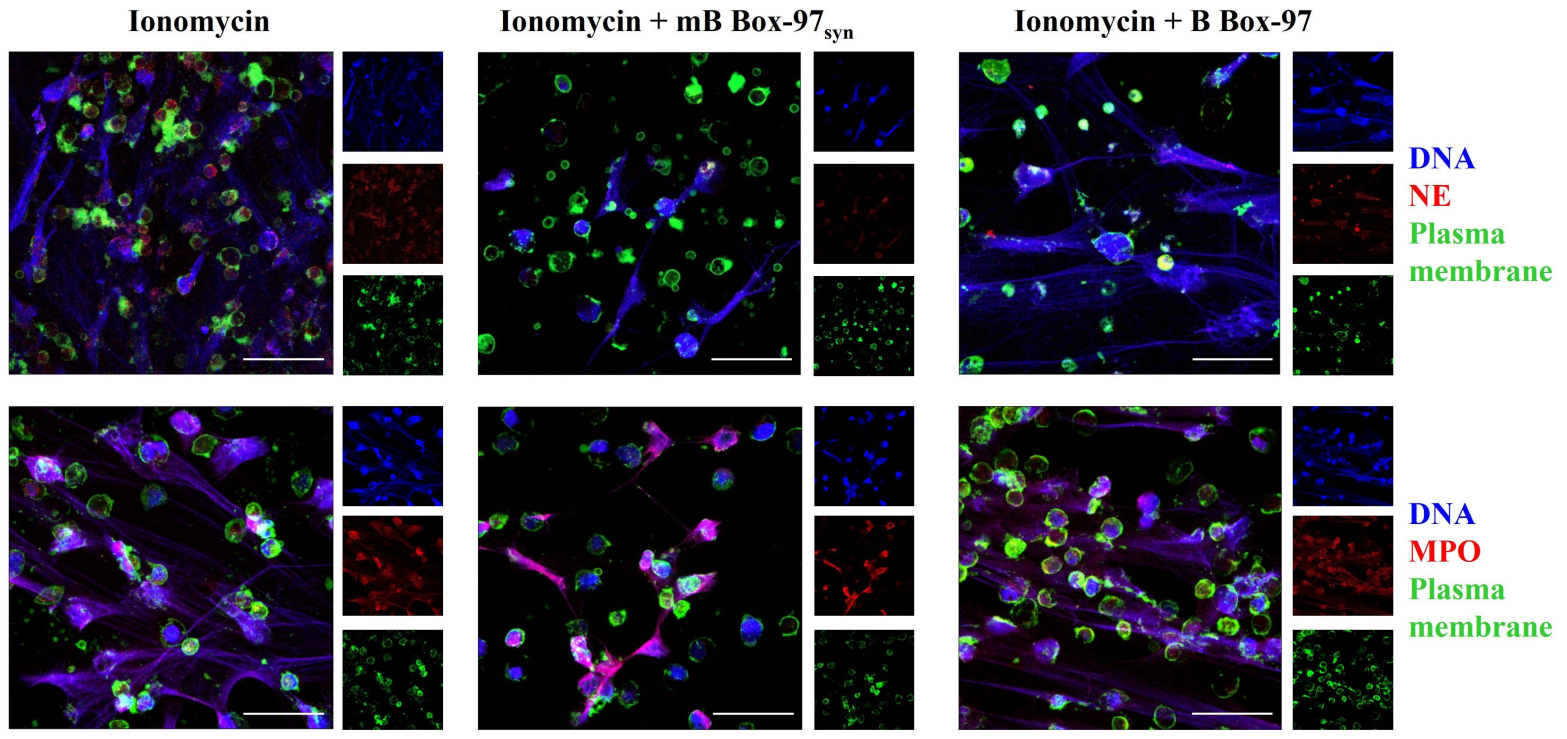
Ca^2+^ ionophore-mediated NETosis was partially inhibited by mB Box-97_syn_. CLSM images of the NETs induced using Ca^2+^ ionophore A23187 with or without mB Box-97_syn_ or B Box-97 are shown where blue represents DNA), green represents plasma membrane, and red represents NE (upper panel) or MPO (lower panel). n = 3 independent experiments, 63x magnification, scale bar 50 µm.

In contrast to the PMA- and LPS-mediated NOX-dependent NETosis, Ca^2+^ ionophore A23187-induced NETosis involves the rapid release of NETs which appears to be independent of NOX activity (1). This Ca^2+-^dependent pathway primarily bypasses PKC-NOX2-ROS mediated-NETosis (1, 2). Therefore, we investigated the effect of mB Box-97_syn_ on Ca^2+^ ionophore-mediated NETosis. Isolated PMNs were treated with A23187 alone or in the presence of mB Box-97_syn_ or B Box-97. NETs were stained as described earlier; mB Box-97_syn_ inhibited Ca^2+^ ionophore-induced NETosis (**Figure 3**), however, the inhibition was not as significant as that observed with PMA/LPS-mediated NETosis (**Figure 2**). MFI of citrullinated histone showed that mB Box-97_syn_ did not have any effect on the histone citrullination upon Ca^2+^-induced NETosis, similar to what was observed upon PMA/LPS-mediated NETosis (**Supplementary Figure S4**). Visual inspection, as well as analysis of MFI value, suggested an increase in the H3Cit staining upon induction with A23187 (**Supplementary Figures S2 & S3C**) which is consistent with the literature (13). mB Box-97_syn_ inhibited Ca^2+^ ionophore-induced NETosis, albeit partially. This result might be due to a rise in Ca^2+^ which can activate PKC (31). These observations prompted us to consider that inhibition of NETosis by mB Box-97_syn_ might be mediated by the PKC/NOX-ROS pathway rather than due to a direct effect on the Ca^2+^-PAD4 pathway.

### 3.4 mB Box-97 localized to the cytoplasm and plasma membrane of human neutrophils

While the inhibitory effect of mB Box-97_syn_ on NETosis was evident, its specific site of action remained unidentified. To gain further insights into its site of action, we investigated the intracellular localization of the peptide within PMNs. To accomplish this, we prepared a recombinant mB Box-97 peptide with an N-terminal His-tag (mB Box-97-His); so as to only track the His-tagged protein and not any of the endogenous HMGB1. mB Box-97-His was found to be stable in serum for at least 24 hours (**Supplementary Figure S5A**). We then assessed the ability of mB Box-97-His to inhibit NETosis (**Supplementary Figure S5B**) and the release of ROS (**Supplementary Figure S5C**). Following confirmation of its inhibitory potency, we conducted a time-course study to elucidate the subcellular distribution of this peptide.

**Figure 4:**
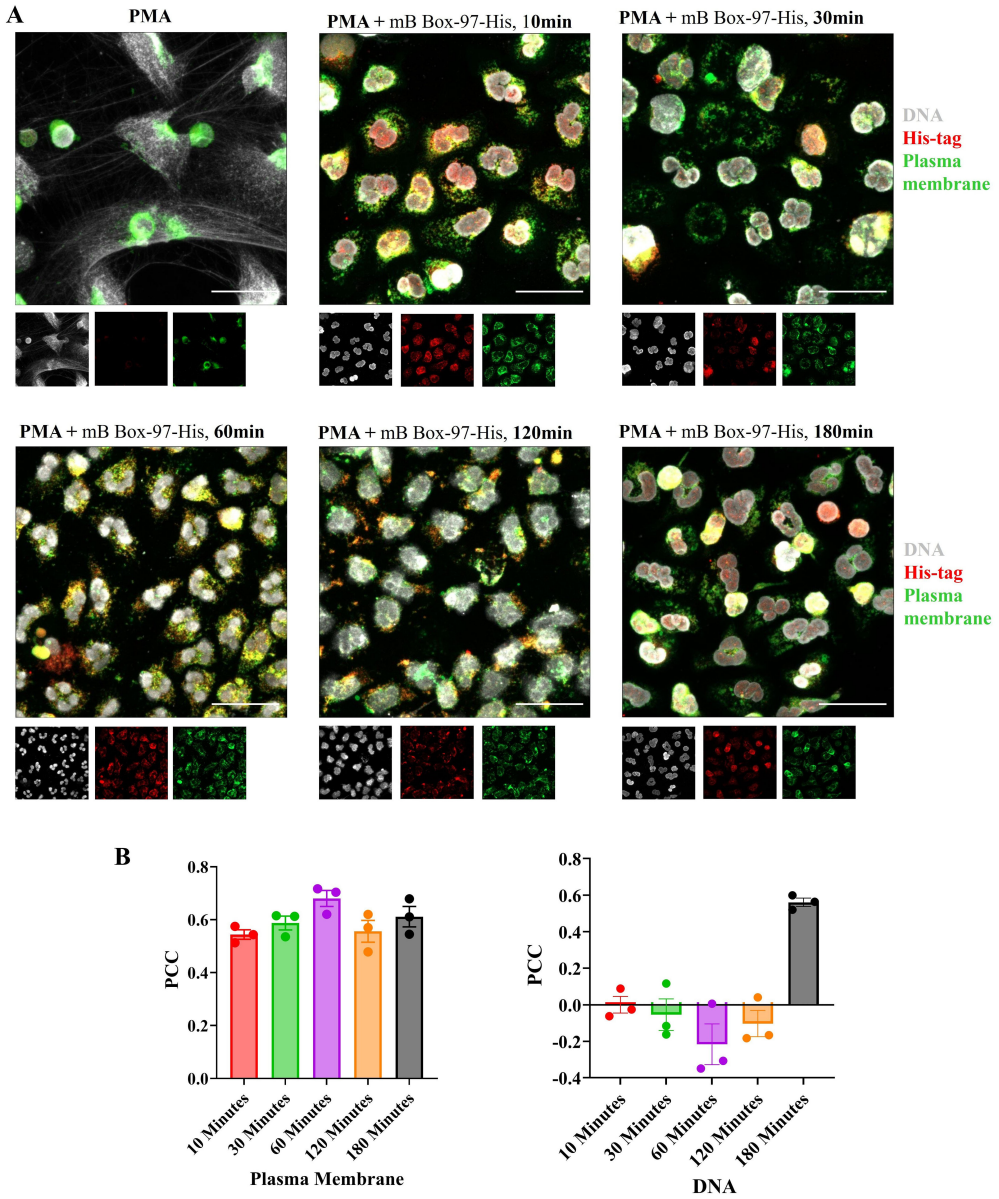
Localization of mB Box-97 within human PMNs. **(A)** Isolated PMNs were exposed to PMA with His-tagged mB Box-97 (mB Box-97-His) for 10, 30, 60, 120, or 180 minutes, or PMA alone for 180 min. Cells were stained to visualize DNA (gray), plasma membrane (green), and mB Box-97-His (red). NETs were visualized by CLSM, with representative 63x images being displayed, with insets at the same magnifications showing the staining for DNA, mB Box-97-His and PM. n = 3 independent experiments, scale bar 50 µm. **(B)** Pearson’s correlation coefficient (PCC) was calculated for the peptide either for the co-localization with PM or DNA for respective treatment time points. Mean values of n = 3 biological replicates ± SEM are shown.

PMNs were incubated with PMA or PMA plus mB Box-97-His for 10 to 180 minutes, fixed, permeabilized and subsequently probed for the presence of the peptide using anti-His antibody. Additionally, DNA and the PM were stained as previously described. Immunofluorescent CLSM images show that the signal corresponding to mB Box-97-His remained robust in the majority of PMNs throughout the tested incubation periods (**Figure 4A**). These images were also analyzed to calculate the Pearson co-localization coefficient (PCC). A PCC of 1 indicates complete co-localization, a value of 0 indicates no specific co-localization and a value of −1 indicates a perfect but inverse correlation (exclusion) (32). PCC values indicate specific co-localization of mB Box-97-His with PM through all time points and with the nucleus at 180 minutes (**Figure 4B**). Additionally, the peptide was consistently detected within the cell cytoplasm across all time points examined (**Figure 4A**). These observations suggest that mB Box-97 can penetrate the PM to get inside PMNs leading to its inhibitory activity.

### 3.5 mB Box-97_syn_ inhibited PKC activity leading to the modulation of p47^phox^ phosphorylation and reduction in ROS production

**Figure 5:**
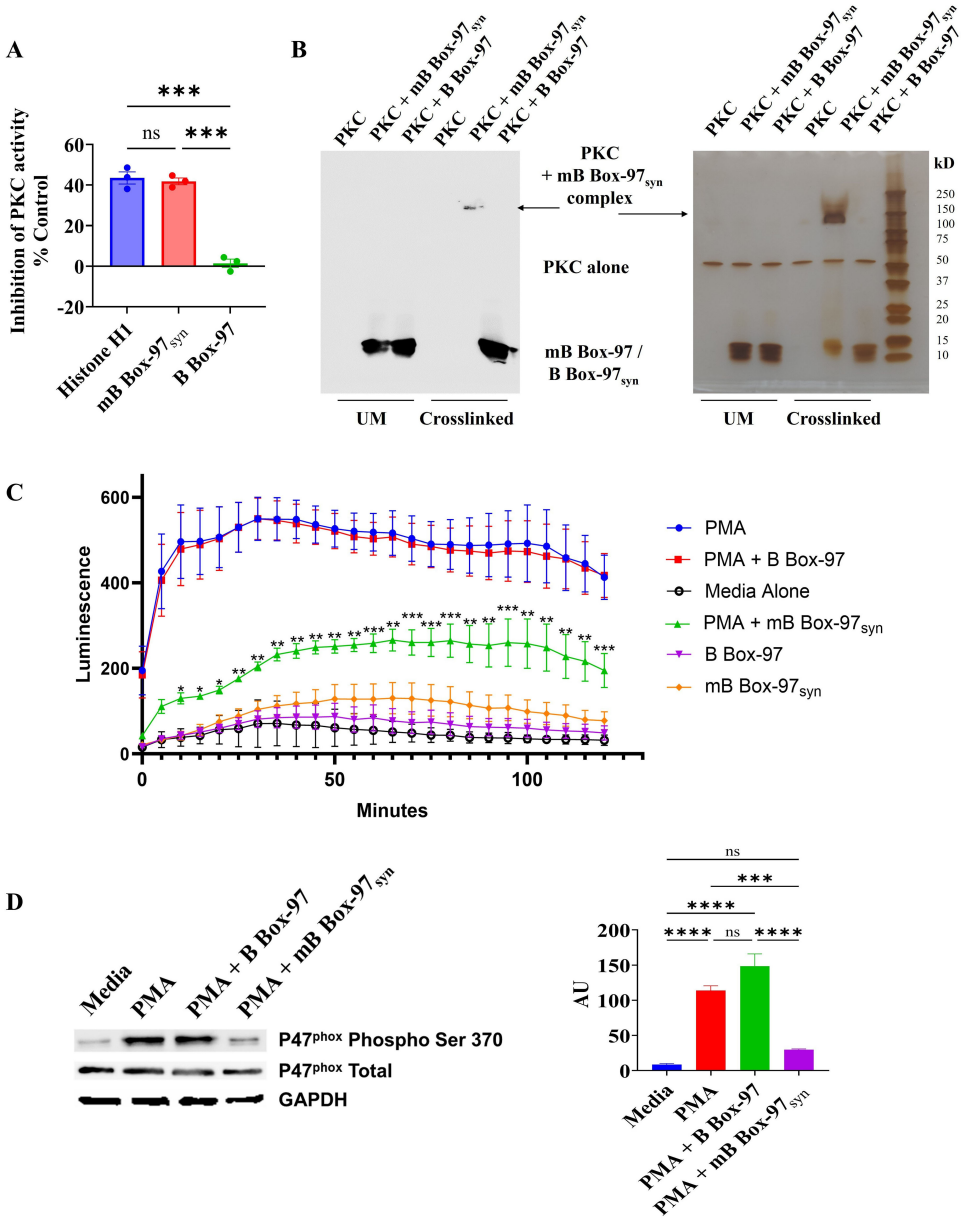
Interaction and inhibition of PKC by mB Box-97_syn_ inhibits P47^phox^ phosphorylation and ROS generation. **(A)** The activity of PKC was assessed using a PKC activity assay kit in the presence of Histone H1, mB Box-97_syn_, and B Box-97. Histone H1, a well-known substrate of PKC, competes with the kit provided PKC substrate peptide used in the assay. As a result, it functions as a competitive inhibitor of PKC and serves as a positive control. Mean values of n = 3 biological replicates ± SEM are shown. **(B)** Conditions unmodified (UM) and crosslinked with glutaraldehyde are indicated. On the left is a Western blot detecting B Box-97/mB Box-97_syn_ and on the right is silver-stained SDS-PAGE separating indicated complexes. PKC crosslinked with mB Box-97_syn,_ PKC alone and B Box-97/mB Box-97_syn_ are indicated. N=3. **(C)** ROS as a measure of Luminol luminescence from PMNs in respective conditions was plotted against incubation time. mB Box-97_syn_ showed a significant reduction in ROS generation compared to PMA alone or PMA + B Box-97 treatment. Mean values of n = 7 biological replicates ± SEM are shown. **(D)** Quantification of steady state levels of phosphorylation of p47^phox^ on Ser-370 in PMNs post treatment under the described conditions was assessed using Western blotting. The intensity of each band was assessed using ImageJ and the relative intensity of phosphorylated p47^phox^ post normalization was plotted as Arbitrary Units (AU). Mean values of n = 4 biological replicates ± SEM are shown. P values (NS >0.05, *p<0.05, **p<0.05, ***p < 0.005, ****p < 0.0001) calculated using Welch and Brown-Forsythe ANOVA followed by Dunnett’s T-test **(A)** or RM two-way ANOVA **(C)** or one-way ANOVA with Tuckey’s multiple t-test **(D)**.

Bioinformatic analysis of mB Box-97_syn_ using the NetPhos – 3.1 tool (33, 34) predicted the introduction of a new PKC phosphorylation site due to a serine residue added through mutation (**Supplementary Figure S6**). Based on our observations of the cytoplasmic localization of mB Box-97_syn_ (**Figure 4**), and the generation of a new PKC phosphorylation site, we investigated whether mB Box-97_syn_ had any effect on PKC activity. As such, we used a PKC activity assay kit to assay whether mB Box-97_syn_ could inhibit PKC kinase activity. Histone H1, a known substrate of PKC (35), was used as a positive control for the inhibition of PKC activity. Indeed, as shown in **Figure 5A** mB Box-97_syn_ but not B Box-97 was an effective inhibitor of PKC.

Next, we examined the binding of PKC to either mB Box-97_syn_ or B Box-97 using glutaraldehyde crosslinking. As shown in **Figure 5B**, mB Box-97_syn_ formed a high molecular weight (~150 kD) complex with PKC. When probed with an anti-HMGB-1 antibody, mB Box-97_syn_ was found in the complex. There was no similar or even additional complex of PKC observed with B Box-97. B Box-97 and mB Box-97_syn_ alone did not show any high molecular weight complex formation in the presence of glutaraldehyde which indicated a lack of any detectable intramolecular interactions (**Supplementary Figure S7**). These observations indicated that PKC is a high-affinity target of mB Box-97_syn_ and the likely means of NETosis inhibition.

Given the importance of ROS in PMA induced NETosis, combined with the membrane-permeable nature of mB Box-97_syn_ that enables its entry into the cell cytoplasm (**Figure 4**), and the ability of the mB Box-97_syn_ to inhibit PKC activity we tested the effect of mB Box-97_syn_ on ROS production by PMNs. Isolated PMNs were preincubated with Luminol, a chemiluminescent indicator of ROS (36). Cells were then activated using PMA alone or PMA with and without B Box-97 or mB Box-97_syn_, or with peptide alone; media alone was used as a negative control. Production of ROS was measured for 2 hours at 5-minute intervals. mB Box-97_syn_ significantly inhibited PMA-induced ROS production in human PMNs (**Figure 5C**). Proteins from treated PMNs were separated by SDS-PAGE and probed for phosphorylation of p47^phox^ on ser-370 with phospho ser-370 specific antibody via Western blotting. Total p47^phox^ and GAPDH were used as controls. Based upon densitometry, mB Box-97_syn_ significantly inhibited the phosphorylation of p47^phox^ while its wildtype counterpart B Box-97 peptide failed to show any similar significant effect (**Figure 5D**).

### 3.6 mB Box-97_syn_ modulated bacterial killing by PMNs

**Figure 6:**
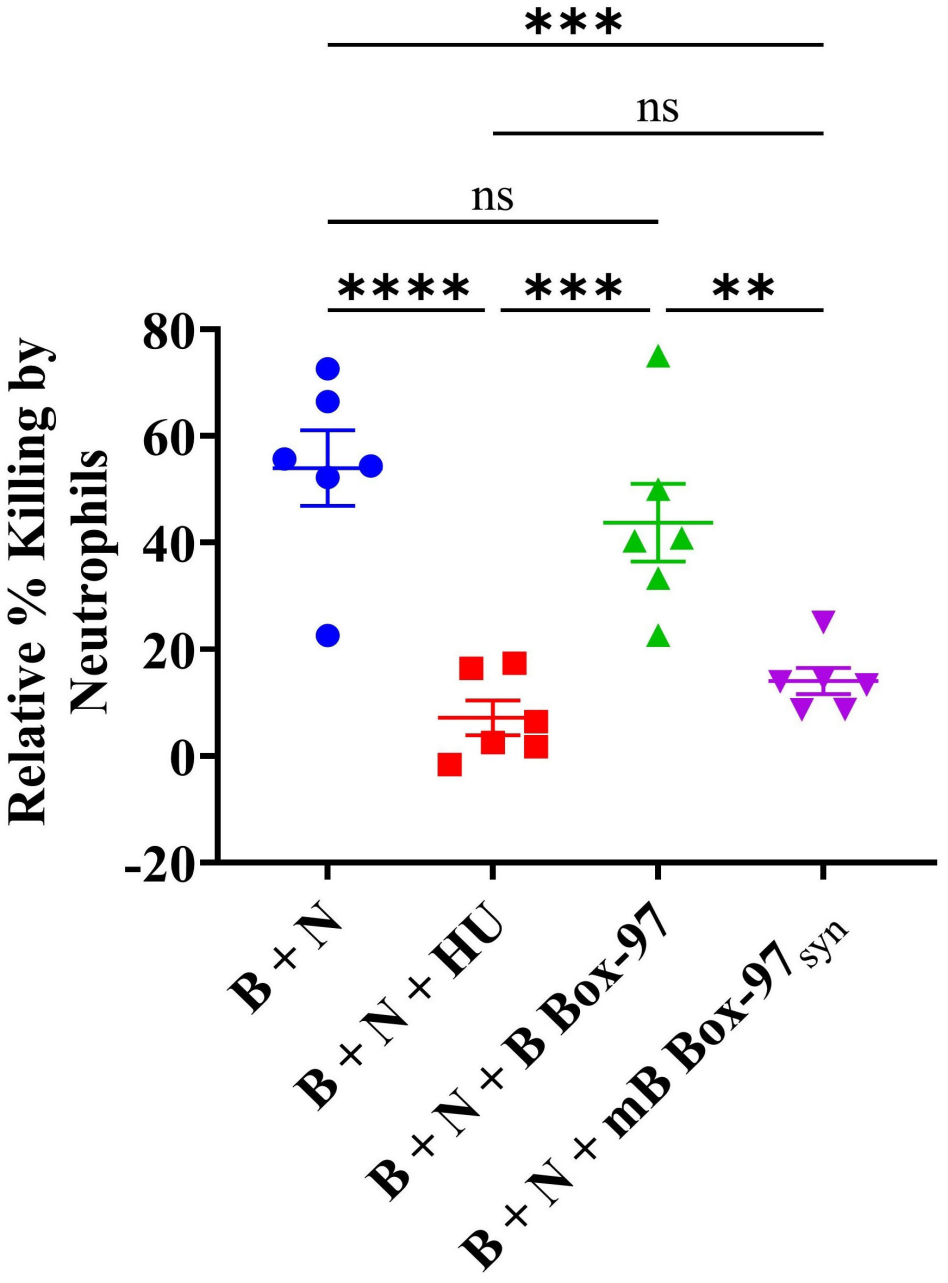
Neutrophil-mediated microbial killing is inactivated by mB Box-97_syn_. NTHI biofilm was incubated with human neutrophils (B + N), or neutrophils treated with recombinant DNABII protein isolated from NTHI (B + N + HU_NTHI_) as a positive control, mB Box-97_syn_ (B + N + mB Box-97_syn_), or B Box-97 (B + N + B Box-97). A control consisting of NTHI biofilm without neutrophil intervention was also included against which the relative percent killing was calculated. Mean values of n = 6 biological replicates ± SEM are shown. P values (**p<0.05, ***p < 0.005, ****p < 0.0001, ns >0.05) were calculated using ordinary two-way ANOVA with Tuckey’s multiple T-test.

As mB Box-97_syn_ was able to inhibit NETosis, PKC activity, ROS production, and release of antimicrobial proteins such as NE, here we tested whether mB Box-97_syn_ could show any downstream effect on bacterial killing. Towards that goal, we incubated PMNs with buffer alone, mB Box-97_syn_, B Box-97, or HU_NTHI_ with a 16-hour NTHI biofilm (where the bacteria are in equilibrium between the young state biofilm and the planktonic state due to natural biofilm remodeling) as described previously (26). We have previously shown that the bacterial DNA-binding protein HU inhibits bacterial killing mediated by NETs (26) therefore we used exogenously supplemented HU here as a positive control. PMNs were lysed with Triton X-100 to recover viable intracellular bacteria, and total CFU NTHI was used to determine the relative percent bacterial killing compared to buffer alone attributable to PMN NETosis (25). mB Box-97_syn_ inhibited bacterial killing by neutrophils (70%) as compared to B Box-97 and this inhibition was similar to that of HU_NTHI_ (80%) (**Figure 6**).

## 4 Discussion

The formation of NETs is an important function of the innate immune system. Studies that show the importance of NETs in the remedy of various pathologies are matched by those that show the negative impact of excessive NET formation (37). Essentially, both the beneficial induction of NETs and their subsequent balance of production and clearance are critical to the beneficial effects of NETs. *In vitro*, various upstream stimulators, including LPS, TNF, IL-8, and PKC agonists, as well as some pre-inflammatory molecules such as HMGB1 can activate NET formation (2, 19, 38). During NETosis various bioactive molecules are released which leads to pathogen killing. However, due to their nonspecific nature, components of NETs can also cause injury to surrounding tissues by either direct action or by increasing the pro-inflammatory response. NETs can also play a role in the enhancement of the inflammation characteristic of autoimmune diseases such as psoriasis, rheumatoid arthritis (RA), and systemic lupus erythematosus (SLE). In addition, autoinflammatory diseases such as gout have been associated with NETosis (4). Excessive NET production can also cause a physical barrier to blood flow which can lead to atherosclerosis and/or stroke (18, 19, 39). Cancer cells can increase NETosis by priming platelets in pancreatic cancer (40). NET-mediated platelet activation can promote several negative outcomes linked with late-stage metastatic breast cancer, including venous thromboembolism (VTE) (41).

Recent studies on SARS-CoV-2 infections (COVID-19) showed that there is a significant role of enhanced neutrophil infiltration and the release of NETs, complement activation, and vascular thrombosis during necroinflammation in COVID-19 (42). Formation of NETs in microvessels increases the inflammatory response and vascular micro-thrombosis, which in the lungs of patients leads to Acute Respiratory Distress Syndrome (ARDS) (43). The serum level of NETosis markers was elevated in patients under intensive care with mechanical ventilation compared to those breathing room air which suggests that NETosis may also be associated with disease severity in COVID-19 (43). Therapeutic inhibition of excessive NET formation may reduce the severity of many such diseases thereby improving survival. Herein, we described an HMGB1-based mutant peptide mB Box-97 which inhibited the formation of NETs induced by multiple stimuli.

There are few direct or indirect inhibitors of NET formation in preclinical studies importantly none of them are currently approved by the FDA for the treatment of NETs-related complications (43). Some of these are molecules that have been in use in clinics for many years such as hydroxychloroquine, methotrexate, and prednisolone (active metabolite of prednisone). Additionally, there are humanized antibodies e.g., rituximab (anti-CD20 mAb), belimumab (fully human IgG1λ recombinant mAb), and Tocilizumab (mAb against IL-6 receptor. In contrast, there are other molecules that can inhibit the function of NE or MPO or PAD4). Many of these compounds function as indirect inhibitors of NETosis (43). The PKC inhibitor ruboxistaurin (LY-333531) has demonstrated anti-NETotic activity against NETs induced by lipopolysaccharide (LPS) by neutrophils isolated from hospitalized patients with COVID-19 (44). Gö 6976, an inhibitor of both PKCα and β, along with LY-333531, a specific inhibitor of PKCβ, exhibit notable reductions in PMA-induced NET formation and ROS production by human neutrophils (12). Although multiple inhibitors are being tested, there is still a need for efficient inhibitors of excessive NETosis that will not elicit off-target collateral damage or other unwanted side effects that could hamper normal beneficial functions of neutrophils.

In our previous work, we showed that an engineered version of HMGB1 (mHMGB1) where we modified Cysteine at amino acid position 45 to Serine, successfully retained its anti-biofilm activity while losing significant proinflammatory properties (23). To further investigate the anti-biofilm potential of HMGB1, we constructed a truncated HMGB1 derived mutated synthetic peptide called mB Box-97_syn_, which retained its anti-biofilm ability against multiple microbes without showing significant pro-inflammatory activity (24). Serendipitously, we observed that this peptide could inhibit NET formation induced by either PMA or LPS while showing partial inhibition of Ca^2+^-induced NET formation, as we described herein.

We also observed the inhibition of ROS generation which is important for the release of antimicrobials from granulocytes as well as the release of decondensed chromatin (45, 46). ROS triggers the dissociation of NE from a membrane-associated complex into the cytosol and activates its proteolytic activity in a myeloperoxidase (MPO)-dependent manner (9). Activated NE moves to the nucleus cleaving chromatin and releasing it into the cytoplasm thereby finalizing the formation of NETs (1, 3). Formation of NETs, release of antimicrobials, and generation of ROS all affect the microbial killing by NETs (3, 47, 48). As mB Box-97_syn_ inhibited ROS generation and release of NETs associated antimicrobial proteins, it was not surprising that there was an effective reduction in neutrophil-mediated bacterial killing in the presence of mB Box-97_syn_.

**Figure 7:**
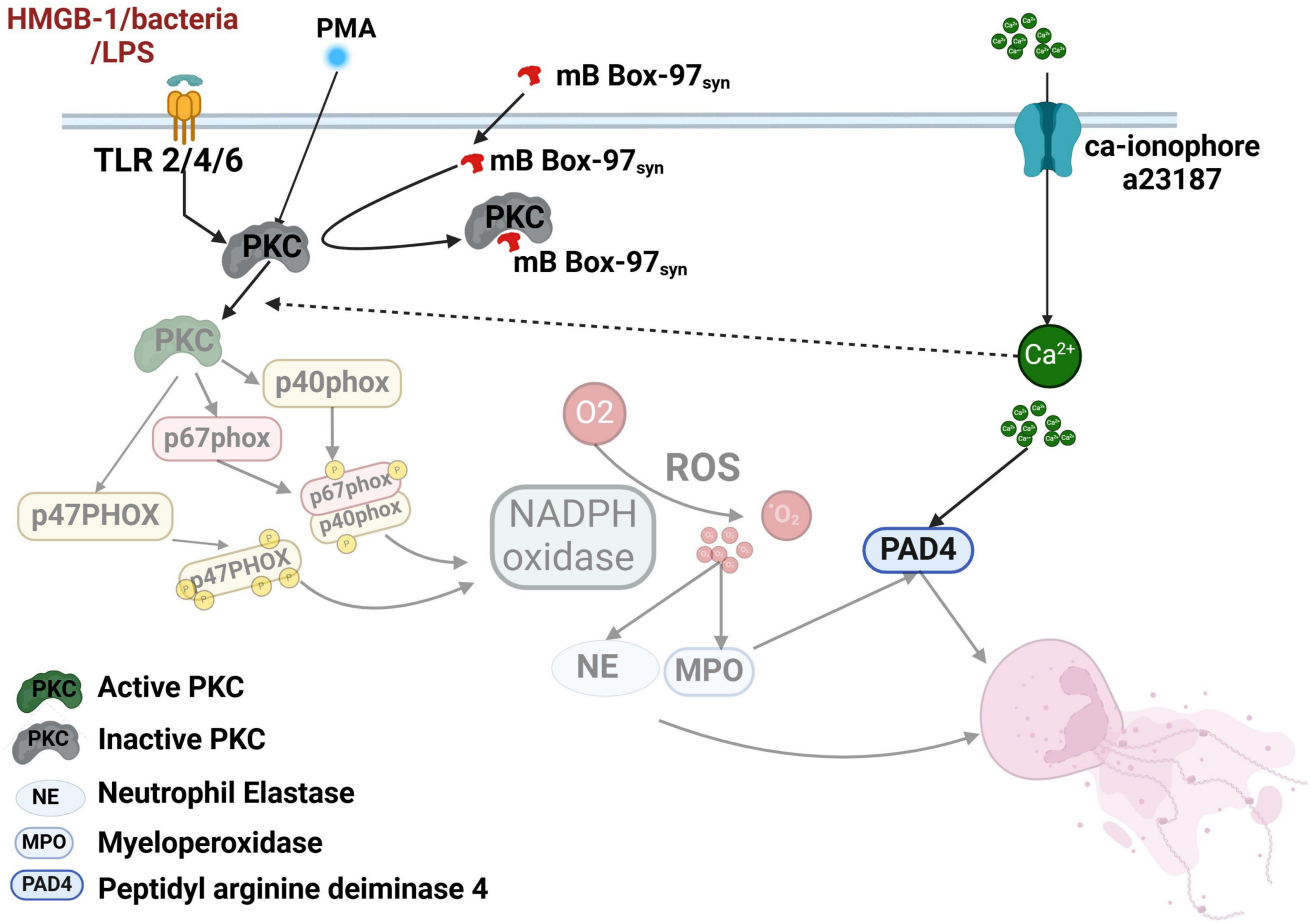
Model summarizing the effect of mB Box-97 on signaling events that lead to NETosis by human neutrophils. The cascade of events affected by mB Box-97 is depicted in a semi-transparent manner. mB Box-97 engages with PKC, thereby inducing inhibition of its enzymatic activity. Consequently, this inhibition impedes the phosphorylation process of p47^phox^, resulting in the suppression of the active assembly of NOX and subsequently reducing the production of ROS. The diminished levels of ROS subsequently lead to a reduction in the release of NE and MPO, ultimately culminating in the inhibition of the formation of the NETs. Furthermore, mB Box-97 demonstrates partial inhibition of Ca^2+^-induced NETosis, plausibly mediated through the suppression of PKC activity, given the influence of Ca^2+^ on PKC functionality.

PKC plays a crucial role in the regulation of NETosis, and its inhibition significantly impairs the process (1, 12, 44, 44). PKC phosphorylates p47^phox^, facilitating its translocation and activation as a subunit of NADPH oxidase (7). Inside the cell, HMGB1 acts as a substrate of PKC which promotes its secretion (49). HMGB1binds to and activates receptors like RAGE (Receptor for Advanced Glycation End Products) and TLR2/TLR4 leading to the formation of NETs (50–52). As HMGB1 acts as a PKC substrate, we tested mB Box-97_syn_ for PKC phosphorylation sites. The mutation of cysteine to serine in mB Box-97, designed to reduce its pro-inflammatory activity, likely introduced a new PKC phosphorylation site. This new serine (106 in the native protein) is located next to an extant serine (107 in the native protein) which suggests that mB Box-97 might be a superior substrate for PKC and compete with PKC for substrates such as p47phox, thereby reducing PKC activity and inhibiting NETosis. PKC, which is inactive in the cytosol, translocates to the plasma membrane upon activation (53, 54). Our co-localization studies show strong co-localization of mB Box-97_syn_ with the plasma membrane. Additionally, while cytoplasmic localization of the peptide could not be definitively confirmed, inhibition of p47^phox^ phosphorylation—a cytosolic event (55) supports the notion that mB Box-97_syn_ enters the cytoplasm. The peptide’s presence at the plasma membrane and its entry into the cytoplasm, where it impairs p47^phox^ phosphorylation, supports a PKC-dependent mechanism for inhibiting NETosis. Indeed, the direct inhibition of PKC activity by mB Box-97_syn_, along with strong protein-protein interactions, confirms that mB Box-97 impedes PKC function. Even the partial inhibition of Ca^2+^-mediated NETosis by mB Box-97_syn_ can be attributed to PKC inhibition, as Ca^2+^ influx can also activate PKC (54), in addition to activating PAD4 (**Figure 7**).

Based on these observations, we postulate that HMGB1-based peptide mB Box-97_syn_ inhibits the activity of PKC which leads to the inhibition of phosphorylation of key NADPH oxidase component proteins. This, in turn, inhibits the active assembly of NOX and reduces ROS production. Reduced ROS production hampers the release of granulocyte-associated proteins like NE and ultimately production of NETs. mB Box-97_syn_ was derived from the native and ubiquitous mammalian protein HMGB1, and while this innate immune effector was engineered to limit inflammation, it retains the ability to inhibit the formation of biofilms (24). These distinct functions are retained in a 97 amino acid contiguous peptide. Future work will determine if the antibiofilm and NETosis prevention functions can be separated to better create therapeutics with singular functions.

## Data Availability

The raw data supporting the conclusions of this article will be made available by the authors, without undue reservation.
