# Supplementary material for: High-mobility group protein B1 derived mutant peptide mB Box-97 inhibits the formation of neutrophil extracellular traps
Source: Front Immunol. 2025 Apr 22;16:1565252. doi: 10.3389/fimmu.2025.1565252 (PMC12059481; doi:10.3389/fimmu.2025.1565252)
Supplement: Supplementary file 1 [file DataSheet1.pdf]

Supplementary figures:

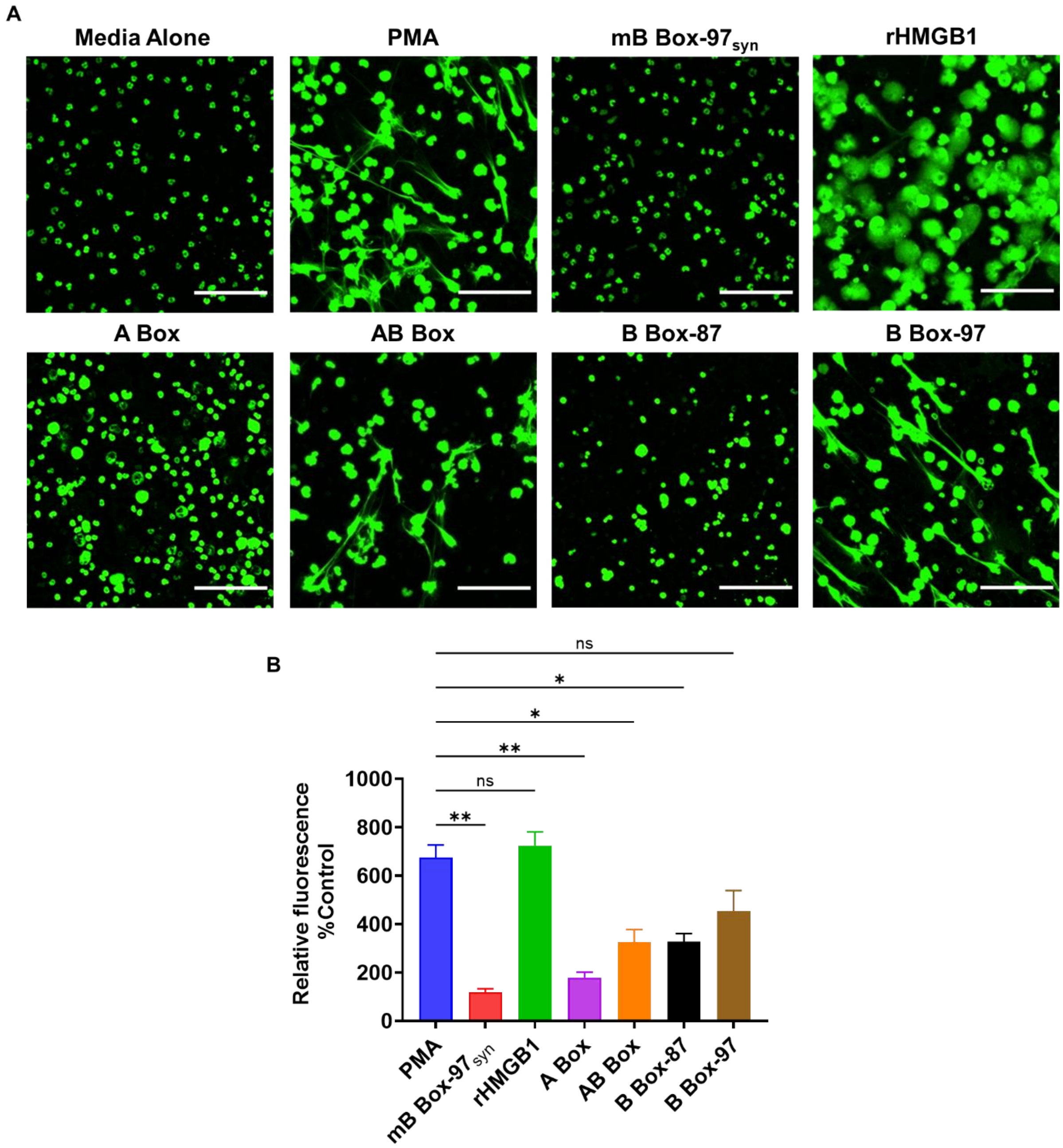

Supplementary Fig. S1. mBBox-97$_{syn}$, BBox-87, A Box, and AB Box do not induce NETosis. **(A)** Isolated human neutrophils were incubated with different HMGB1-derived peptides; A Box, B Box-87, AB Box, B Box-97, mB Box-97$_{syn}$ or recombinant HMGB1 (rHMGB1), media alone was used as a negative and PMA as a positive control. After incubation neutrophils were fixed, and DNA was stained with SYTOX^{TM} green and NETs visualized by CLSM at 20x magnification, and representative confocal microscopy images were shown out of 3 independent

experiments (n), scale bar 50 µm. **(B)** Nets were induced in isolated neutrophils using respective constructs as above and released DNA was quantified using cell impermeable dye SYTOX™ green using a fluorimeter. % relative fluorescence compared to media alone control is plotted. Mean values of $n = 5$ biological replicates ± SEM are shown. p values (ns = $p > 0.05$, ****$p < 0.0001$) are from a Welch and Brown-Forsythe ANOVA followed by Dunnett's T test.

A

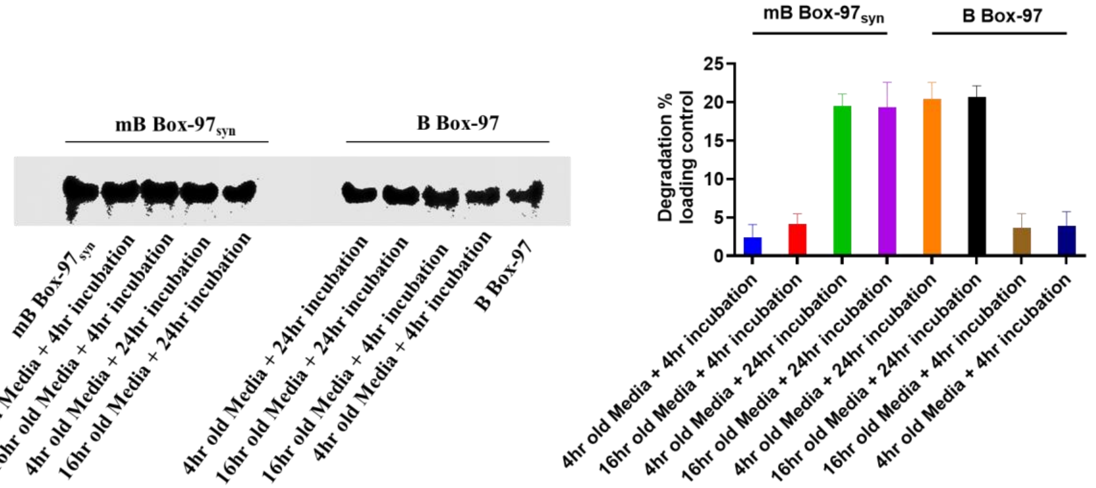
mB Box-97syn
B Box-97
mB Box-97 syn
4hr old Media + 4hr incubation
16hr old Media + 4hr incubation
4hr old Media + 24hr incubation
16hr old Media + 24hr incubation
4hr old Media + 24hr incubation
16hr old Media + 24hr incubation
16hr old Media + 4hr incubation
4hr old Media + 4hr incubation
B Box-97
Degradation % loading control
0
5
10
15
20
25

B

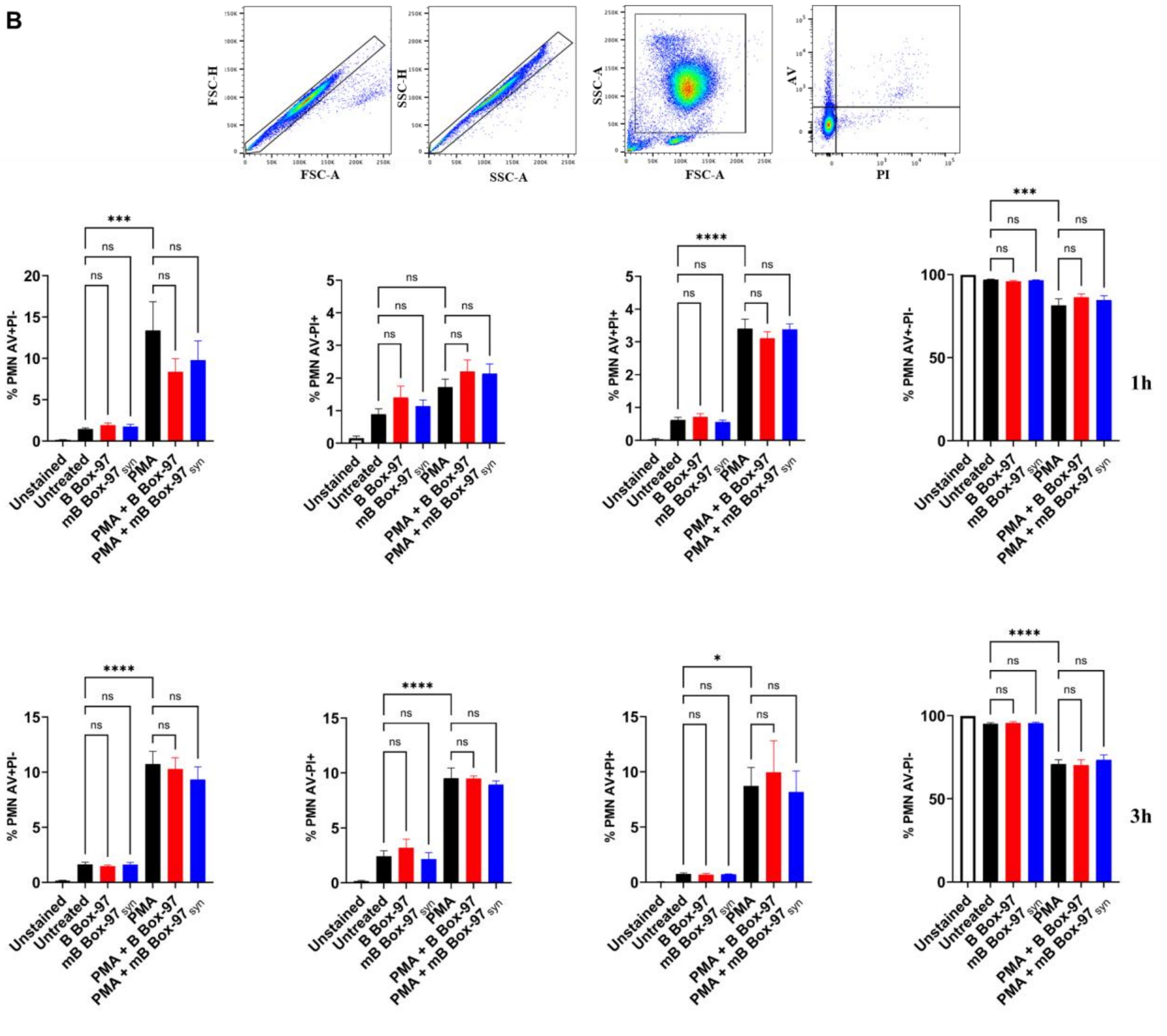
FSC-H
FSC-A
SSC-H
SSC-A
SSC-A
FSC-A
AV
PI
% PMN AV+PI-
% PMN AV-PI+
% PMN AV+PI+
% PMN AV-PI-
Unstained
Untreated
B Box-97
mB Box-97 syn
PMA
PMA + B Box-97
PMA + mB Box-97 syn
ns
*
***
****
1h
3h

Supplementary Fig.2. mB Box-97$_{syn}$ is stable in the Neutrophil media and does not affect neutrophil viability. (A) Representative western blot image showing mB Box-97$_{syn}$ and B Box-97 post incubation for 4 and 24 hours at 37°C with conditioned media collected from the neutrophils which were induced to form NETs using PMA for 4 and 16 hours. The graph shows the quantification of degradation % compared to the loading control of 4 independent experiments, N=4. **(B)** Peripheral blood neutrophils were treated with mB Box-97$_{syn}$ or B Box-97, either alone or in combination with PMA in HBSS media. The cells were collected at 1 and 3 hours post stimulation and subsequently stained with anti-Annexin V (AV) FITC antibody and Propidium Iodide (PI) to assess cell viability. Viable cells were separated by Flow cytometry. The gating strategy involved the selection of single-cell events, followed by classification into cells positive for Annexin V (early apoptotic cells), PI-positive (late apoptotic/necrotic, including NETotic neutrophils), double positive, or double negative. The bar graphs show the percentage of total neutrophils in each quadrant. No significant differences were observed between untreated neutrophils and those treated with B Box-97 or mB Box-97$_{syn}$ or neutrophils treated with PMA alone or with peptides. The statistical analysis was performed using ordinary one-way ANOVA and Tukey's multiple comparison test (n = 3 donors, ns = $p > 0.05$, *=$p<0.05$, ***=$p<0.001$, ****=$p<0.0001$).

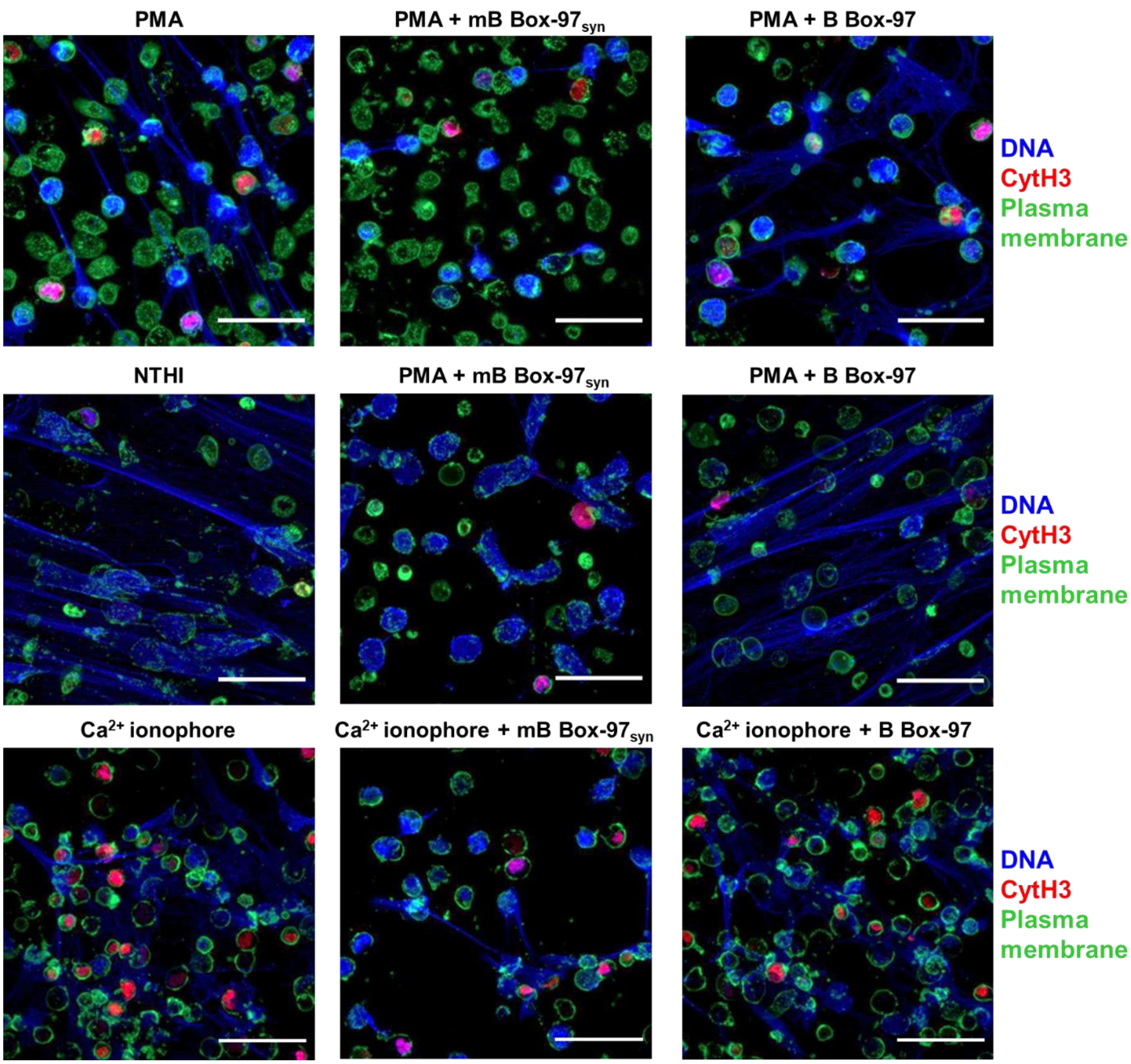

Supplementary Fig.3. Detection of Citrullinated histone H3 (H3Cit) in NETs induced with various stimuli. Isolated PMNs were allowed to form NETs in the presence of PMA/NTHi/ Ionomycin with or without B Box-97/mB Box-97$_{syn}$. The presence of H3Cit (red) was investigated using confocal microscopy and the representative images were shown. N=3, scale bar 50 µm.

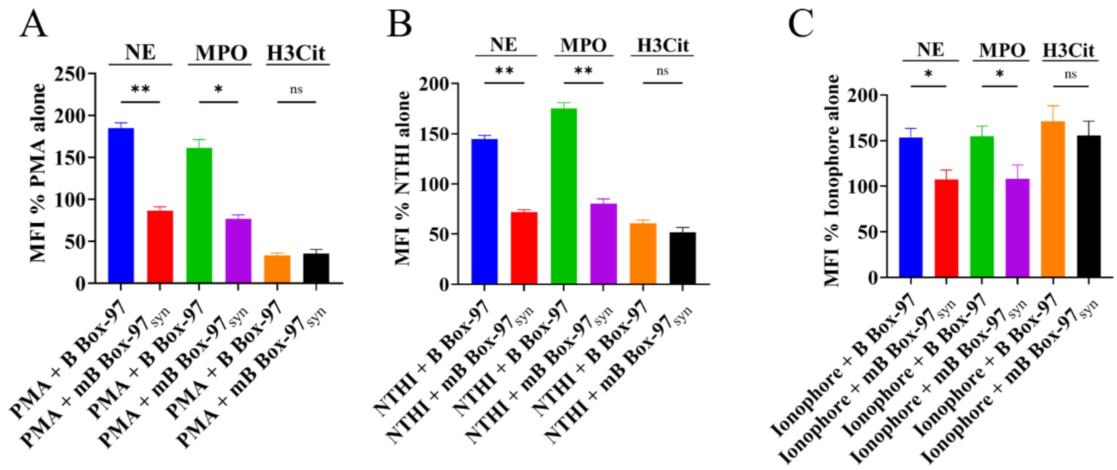

Supplementary Fig.4. Changes in Mean Fluorescence Intensity (MFI) of the NETs associated proteins. MFI values for NE/MPO/H3Cit were normalized with MFI for dsDNA and PM and plotted as a % of **(A)** PMA alone, **(B)** NTHI alone and **(C)** Ca^{2+} ionophore alone treated control. Mean values of $n = 3$ biological replicates ± SEM are shown. p values (ns = $p > 0.05$, $*p < 0.05$, $**p < 0.01$) are from a Welch and Brown-Forsythe ANOVA followed by Dunnett's T test.

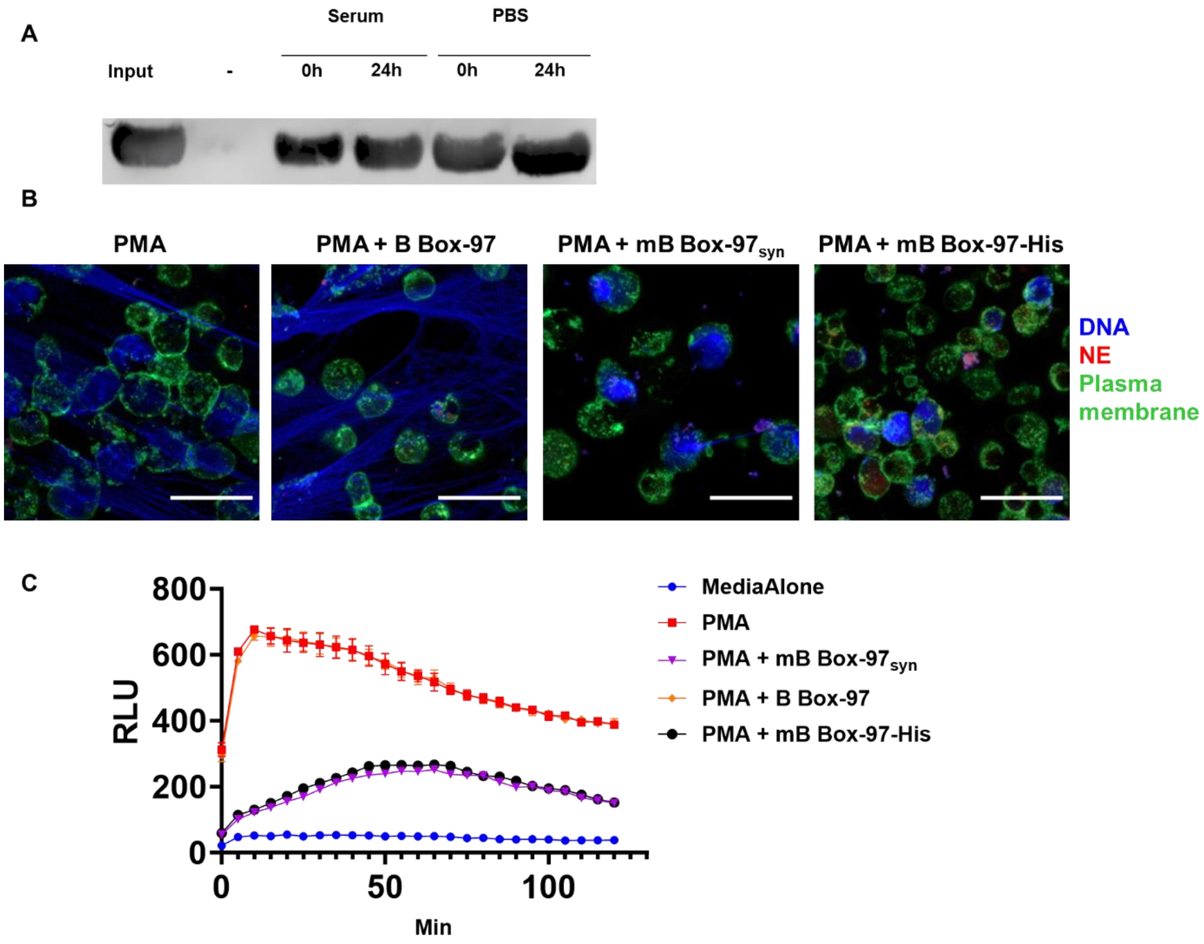

Supplementary Fig.5. mB Box-97-His is stable in the serum and shows comparable inhibition of NETosis and ROS generation to mB Box-97$_{syn}$. (A) western blot showing mB Box-97-His incubated for 24hr in 50% human serum and PBS at 37°C. **(B)** CLSM images of neutrophils induced with PMA with or without B Box-97/mB Box-97$_{syn}$ / mB Box-97-His. DNA is shown in blue, NE in red and PM in green, N=3, scale bar 50 μm. **(C)** ROS as a measure of Luminol luminescence from PMNs in respective conditions was plotted against incubation time. Mean values of n = 4 biological replicates ± SEM are shown.

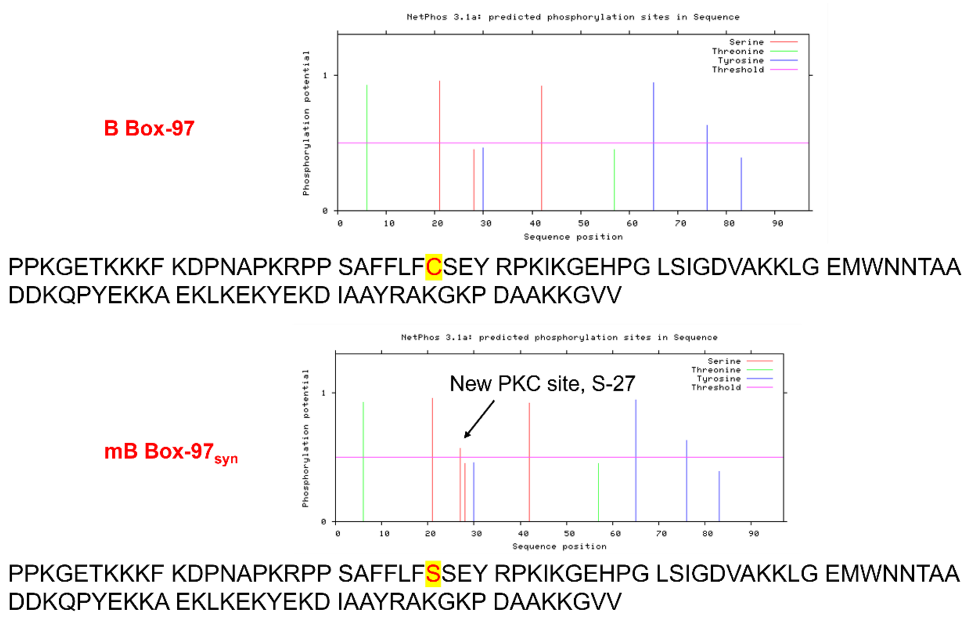

Supplementary Fig.6. Identification of PKC phosphorylation sites on mB Box-97$_{syn}$. New PKC phosphorylation site at S-27 (mutated from corresponding C of B Box-97) (shown by arrow) on mB Box-97 as predicted by online prediction tool NetPhos - 3.1. Sequences for respective peptides are shown below each peptide with the mutant residue highlighted in yellow and shown in red.

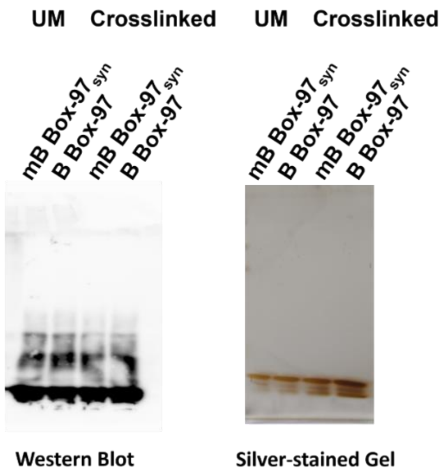

Supplementary Fig.7. Effect of glutaraldehyde on mB Box-97$_{syn}$ or B Box-97. The formation of a multiprotein complex of mB Box-97$_{syn}$ and B Box-97 in the presence of glutaraldehyde was tested. UM is unmodified and cross-linked with glutaraldehyde are indicated. On the left is a western blot detecting mB Box-97$_{syn}$ /B Box-97 and on the right is silver-stained SDS-PAGE gel.
